# Fly or Dry? River Flow and Dispersal Mode Drive Cross‐Channel β Diversity in Riparian Zones

**DOI:** 10.1002/ece3.73568

**Published:** 2026-05-04

**Authors:** Kieran J. Gething, Romain Sarremejane, Chloe Hayes, Jaime Martin, Robert I. Collier, Jonathan R. Webb, Judy England, Tim Sykes, Rachel Stubbington

**Affiliations:** ^1^ Loughborough University Loughborough UK; ^2^ Nottingham Trent University Nottingham UK; ^3^ Environment Agency Bristol UK; ^4^ Natural England Peterborough UK

**Keywords:** connectivity, contraction, drying, flow permanence, habitat fragmentation, river

## Abstract

Terrestrial arthropods form biodiverse communities that support the structure and functioning of riparian ecosystems and are shaped by local to large‐scale connectivity in three dimensions. In particular, river characteristics such as size and flow permanence are likely to influence lateral cross‐channel connectivity, but how such temporally variable natural barriers shape riparian communities is rarely quantified. We tested the connectivity of communities comprising a diverse family of largely ground‐dwelling terrestrial arthropods (ground beetles, Coleoptera: Carabidae) by repeatedly sampling parallel riparian zones along rivers spanning a gradient of flow permanence during flow recession—when declining water levels theoretically reduced the barrier separating communities on left and right banks. We quantified cross‐channel connectivity by comparing left–right bank community dissimilarity using Sørensen β diversity and null‐model‐derived z‐scores, distinguishing species capable of flight from those with limited or no flight ability. Communities in parallel riparian zones were similar along the gradient, suggesting that river channels represent comparably weak barriers to cross‐channel movement regardless of water levels. In particular, species capable of flight were unaffected by in‐channel conditions, whereas the cross‐channel comparability of assemblages with limited flight abilities increased as flows declined. The replacement of water‐associated species by generalists as flows declined suggests that access to water as a resource may be more important than the barrier posed by water in structuring riparian ground beetle communities. To balance access to key resources, such as water, with the connectivity communities require for long‐term resilience, management actions should seek to mitigate climate‐driven shifts in spatiotemporal extent of river drying.

## Graphical abstract

Terrestrial arthropods play a key role in riparian ecosystems, but it remains unclear whether drying stream channels act as barriers shaping communities on opposite banks. By sampling a family of largely ground‐dwelling arthropods along a gradient of flow permanence during a flow recession, we found that communities on parallel banks were generally similar, with flying species being unaffected by channel conditions and assemblages of limited‐flight species becoming more similar as water levels declined. These findings suggest that access to water as a resource may structure riparian arthropod communities more strongly than the physical barrier of the channel itself, highlighting the importance of river drying in maintaining ecological connectivity and resilience.

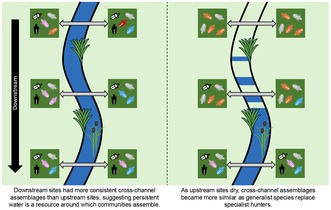

## 1 Introduction

Terrestrial arthropods are a ubiquitous and biodiverse component of ecosystems worldwide (Stork 2018). The distribution of terrestrial arthropods is influenced by large topographical features (e.g., large rivers, mountains: Satler and Carstens 2017; Musthafa et al. 2021), which can impede movement and colonisation, resulting in reduced connectivity and compromising community resilience to extreme events such as drought or floods (Van Looy et al. 2019). Beyond these large‐scale features, smaller barriers such as river channels may also influence arthropod movement, with their influence changing through time, e.g., as flows fluctuate. However, the role of smaller, temporally variable natural barriers in shaping arthropod communities is rarely quantified (Sánchez‐Montoya et al. 2023).

Most of the world's rivers sometimes dry out (Messager et al. 2021), and the spatial and temporal extent of river drying is increasing in many global regions (Zipper et al. 2021; Mimeau et al. 2025). During dry phases, river channels can be colonised by diverse and abundant assemblages of riparian arthropods (e.g., ground beetles, spiders: Steward et al. 2011; Corti and Datry 2016; Gething 2024). In contrast, wetted rivers may represent barriers between communities in parallel riparian zones (i.e., the riverine barrier hypothesis: Wallace 1854; Sánchez‐Montoya et al. 2023), with an arthropod's capacity to disperse across the channel and thus maintain connectivity between communities on parallel (i.e., left and right) banks (hereafter, cross‐channel communities) being determined by its inundation tolerance (Kolesnikov et al. 2012), dispersal mode (e.g., ground‐based, aerial: Ružanović et al. 2025), and river hydrology and morphology (e.g., the width, depth and velocity of the water: Lindroth 1992; Carrasco et al. 2022).

Narrow, linear habitat features with sparse surface vegetation (e.g., roads, railways) can be barriers to ground‐based and aerial arthropod movement (Mader 1984; Lövei et al. 1998; Andersson et al. 2017), encouraging organisms to move along rather than across such features (Mader et al. 1990). Both wet and dry river channels may have similar effects, with the extent of ground‐based movement across the channel likely being influenced by in‐channel conditions (Lindroth 1992). Although water is no physical barrier to aerial dispersers, it might be a perceptual barrier (Lövei et al. 1998; Andersson et al. 2017). In addition, when individuals do disperse by flight it is energy intensive, dependent upon life stage (Matalin 1994), and influenced by environmental conditions (e.g., weather: Heino and Alahuhta 2015) and the relative favourability of the source and surrounding habitats (Lövei and Sunderland 1996).

Riparian arthropods in unfavourable habitats move further and more frequently than those experiencing favourable conditions (Lövei and Sunderland 1996). Thus, although energy expenditure and linear habitat features may limit lateral movement, the relative favourability of habitat conditions may trigger organisms to disperse into, and ultimately across, river channels (Ružanović et al. 2025). For example, drying temporary reaches often retain some water, which is associated with food resources such as stranded aquatic animals (O'Callaghan et al. 2013; Freixinos et al. 2024, 2025), and may contain fewer competitors than nearby riparian habitats (Sánchez‐Montoya et al. 2020). Drying river habitats may thus offer more resource opportunities than riparian zones, encouraging movement into the channel (Steward et al. 2022; Ružanović et al. 2025). Factors including the temporal stability of in‐channel conditions, the extent of any remaining water and an individual's capacity for movement thus determine any subsequent movement from the channel into either their original or the opposite riparian zone.

Riparian arthropod communities exhibit strong lateral zonation around rivers (i.e., different species prefer to live at different distances from the river: Paetzold et al. 2005; Bates et al. 2007; Sánchez‐Montoya et al. 2016), potentially driven by species‐specific inundation tolerances (O'Callaghan et al. 2013; Gething et al. 2026). Additionally, longitudinal changes in river flow (and covarying factors, e.g., sediment and vegetation characteristics) influence community composition (e.g., McCluney and Sabo 2012; Sánchez‐Montoya et al. 2016), with riparian zones in headwater reaches largely supporting habitat generalists (*sensu* Gooderham et al. 2007; Devictor et al. 2008), whereas specialists become more common with distance downstream (Eyre, Lott, and Luff 2001; Eyre, Luff, and Phillips 2001; Gething et al. 2026). Assemblages in dry river channels may also exhibit spatial zonation, but this varies through time (e.g., Sánchez‐Montoya et al. 2020) because the lateral and longitudinal extent of water varies across diel to multi‐year timescales (e.g., Burt 1979; Claret and Boulton 2003; Bunting et al. 2021).

In the riparian zones of river reaches with perennial flow, microhabitats such as riparian vegetation and gravel banks, and food resources including emerging aquatic insects, are relatively stable over time, allowing terrestrial arthropod communities to specialise (Hering et al. 2004; Paetzold et al. 2005; Ramey and Richardson 2017). Although generally temporally stable on each bank, the distribution of habitats and resources may differ between parallel riparian zones, for example if land uses differ. Thus, the combination of a permanent barrier—water—that promotes longitudinal rather than lateral movements (Mader et al. 1990) and the potentially different habitats and associated resources on parallel banks may discourage or prevent cross‐channel movement in perennial reaches, especially by ground‐dwelling organisms (Braaker et al. 2017). Such isolation and specialisation to site‐specific conditions can result in greater spatial variability in community composition, i.e., higher spatial β diversity (Hubbell 2001; Sánchez‐Montoya et al. 2020; Hu et al. 2022). In contrast, partial or complete river drying may temporarily increase connectivity between communities in parallel riparian zones. Such connectivity may reduce the spatial β diversity of cross‐channel communities, increasing resilience, but may also lead to communities dominated by mobile, generalist species that can exploit new in‐channel and cross‐channel opportunities (Eyre, Lott, and Luff 2001; Eyre, Luff, and Phillips 2001; Gooderham et al. 2007).

As river drying becomes more common (Zipper et al. 2021; Mimeau et al. 2025), we aimed to assess the role of flow permanence gradients in shaping riparian arthropod communities. We selected ground beetles (Coleoptera: Carabidae) to represent riparian arthropods because they are a common, species‐rich family in riparian ecosystems (e.g., Sadler et al. 2004; Steward et al. 2011; Bunting et al. 2021), have well‐known environmental preferences, and include species with ground‐based and aerial dispersal modes (Luff 2007; Knapp et al. 2020). We hypothesised that the cross‐channel β diversity of riparian ground beetle communities decreases: (H1) from downstream to upstream, due to greater opportunities for mixing across dry and drying channels; and (H2) as the river dries, because the decreasing water width and depth represent a diminishing barrier to cross‐channel movement.

## 2 Materials and Methods

### 2.1 Study Area

**FIGURE 1:**
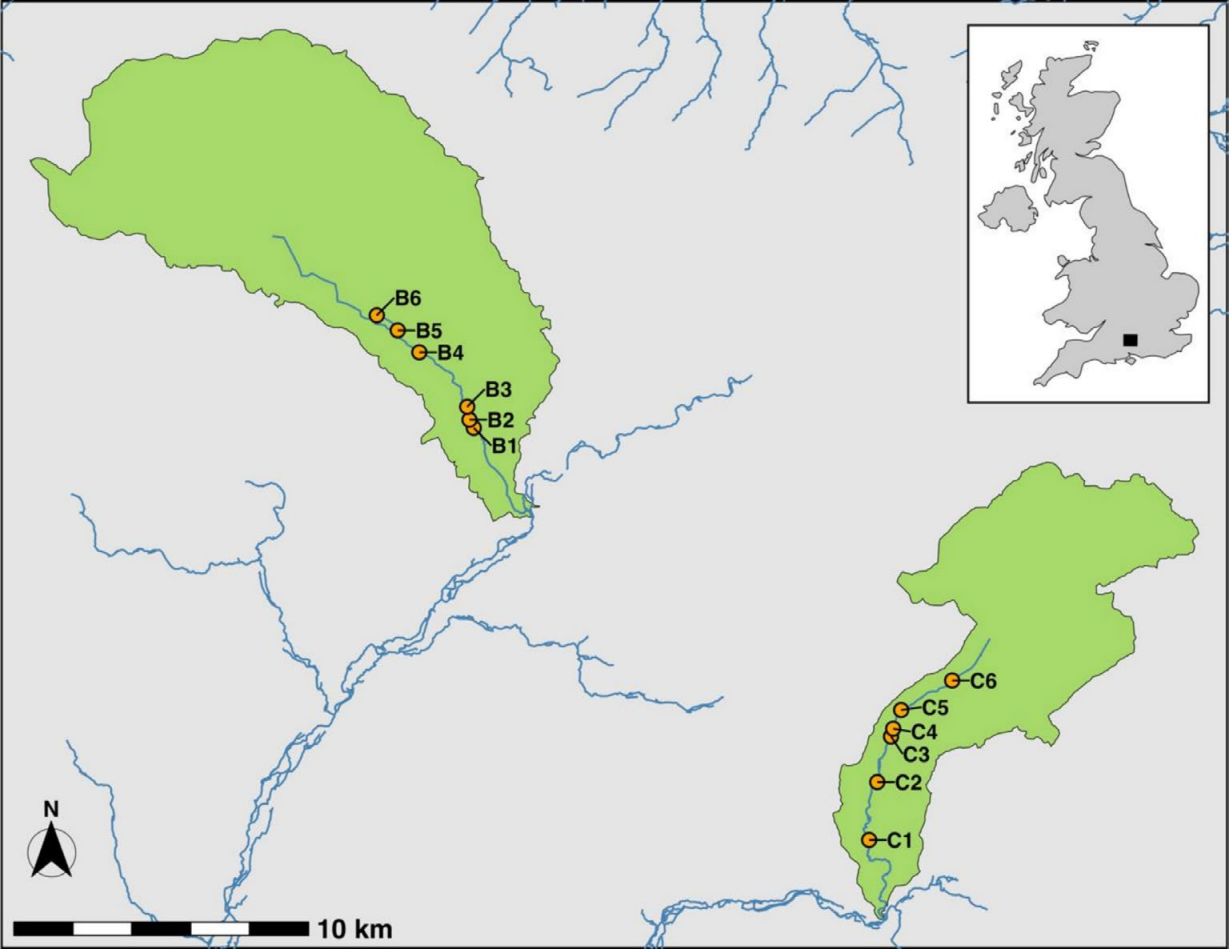
Sampling sites in the Bourne Rivulet (west) and Candover Brook (east) catchments. Site codes indicate the river name (Bourne, B; Candover, C), and the longitudinal position from downstream to upstream (1–6).

We studied the riparian zones of the Candover Brook and the Bourne Rivulet, Hampshire, UK (Figure 1). The area has a temperate oceanic climate (Cfb: Kottek et al. 2006), with annual minimum and maximum air temperatures of 6.1°C ± 3.9°C and 15.0°C ± 5.7°C (mean ± SD), respectively, and mean annual rainfall of 754 mm (Met Office 2022). Land use in both catchments is dominated by arable agriculture (> 50%, National River Flow Archive [NRFA] 2021) and pasture (> 28%) with few urban areas (< 5%). The catchments are underlain by a chalk aquifer, which causes upstream‐to‐downstream drying in the headwaters of both rivers during summer when groundwater levels are low (Sear et al. 1999).

Six sampling sites along each river spanned 7.3 km and 5.6 km of the Candover Brook and the Bourne Rivulet, respectively (Figure 1). On each river, the sites spanned a flow permanence gradient in which, in a typical year, Site 1 was perennial and Site 6 flowed for ~3 months. Five sites on each river had comparable land uses (mown grassland, pasture or wetland: Table S1) on both banks and extending ≥ 6 m laterally from the water's edge, including the bank face and ≥ 4 m of the adjacent terrestrial land. Site B2 had woodland on one bank and mown grassland on the other, and site C5 had wet woodland on one bank and a mown grass verge with a road parallel to the channel at a distance of ~4 m on the other.

### 2.2 Study Design and Sampling

**FIGURE 2:**
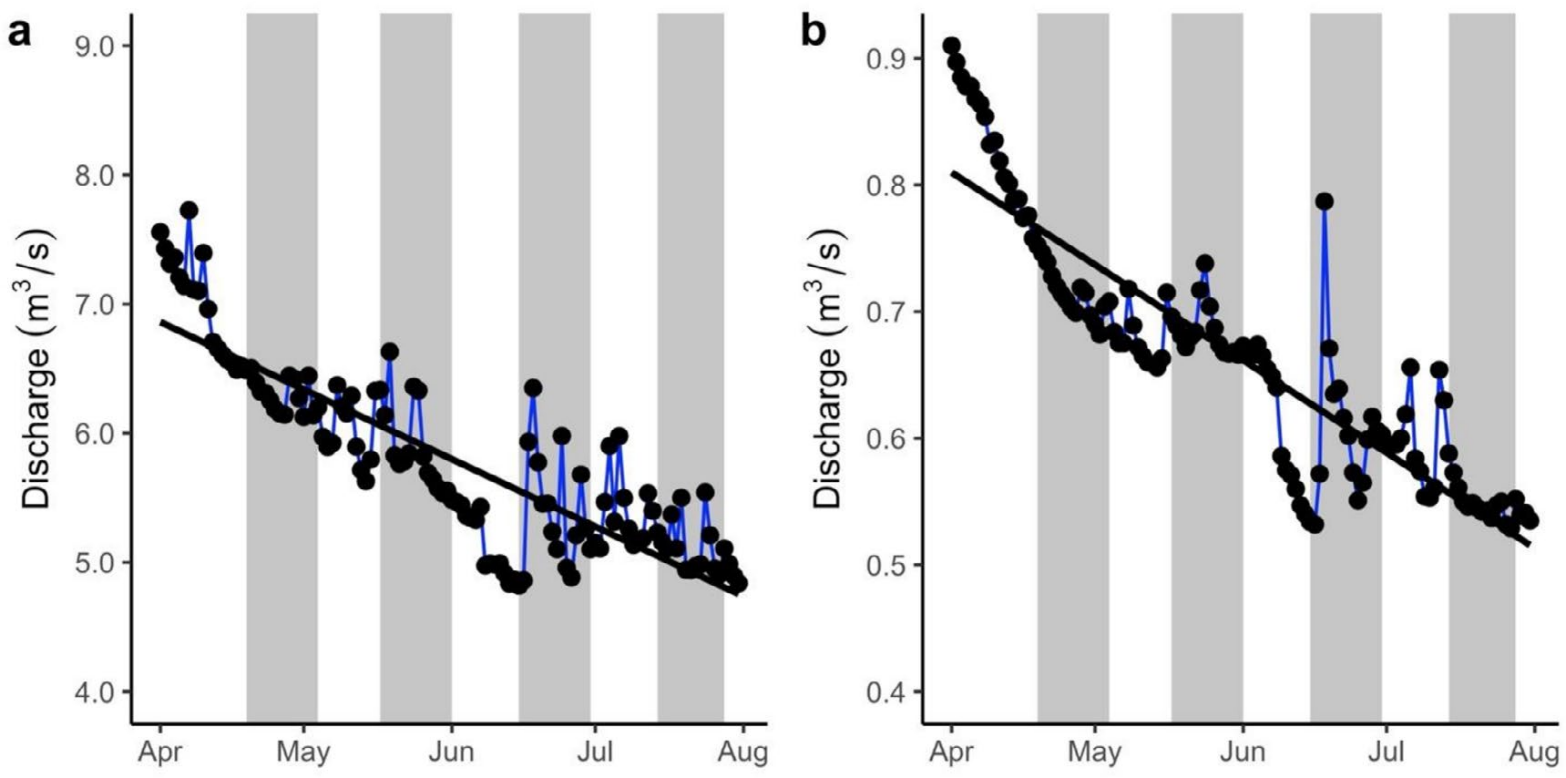
Daily discharge during the study period at the downstream gauging station closest to the study reaches on the Bourne Rivulet (a) and Candover Brook (b). Circles represent discharge and are linked by blue lines representing changes in discharge between consecutive days, black lines represent the trend in discharge over the study period and grey shaded areas represent sampling periods. Data sources: NRFA (2022a, 2022b).

We sampled all sites during four equally spaced visits (V1–4) between mid‐April (spring) and mid‐July (summer) 2021 (see sampling periods on Figure 2), i.e., encompassing (and extending 2 weeks beyond) the main April**–**June carabid survey season (Drake et al. 2007). We made observations of the width and depth of water in the channel at each site relative to previous visits. We used discharge data from the closest gauging station on each river, both of which are located on downstream reaches, to supplement these observations. The Bourne Rivulet gauging station was located approx. 12 km downstream of B1 on the River Test (NRFA 2022a) and the Candover Brook station was 3 km downstream of C1 (NRFA 2022b).

We sampled invertebrates by pitfall trapping, broadly following Drake et al. (2007) and Webb et al. (2022). At each site, we set six pitfall traps on each side of the channel at 2‐m intervals. Traps were 0.25–3 m from the water's edge during V1. We 1/3 filled the cups with an ethylene glycol–water mix to preserve trapped invertebrates. After a 15‐ to 16‐day sampling period, we collected and pooled the six traps from each side of the channel, creating one left and one right bank sample. In total, we set 576 pitfall traps (2 rivers × 6 sites per river × 2 samples per site × 6 pitfall traps per sample × 4 dates) and pooled them into 96 samples. We discounted both B6 V4 samples due to disturbance by livestock, leaving 94 samples.

### 2.3 Data Preparation and Analysis

We identified ground beetles mostly to species or a species aggregate, with 1.0% and 0.2% of individuals being identified to genus and family, respectively. To avoid the inflation of cross‐channel variation by taxa identified to multiple taxonomic levels (e.g., *Pterostichus madidus* and *Pterostichus*), we assigned genera to a single most‐likely species/species aggregate (*sensu* Cuffney et al. 2007). Because the similarity of communities on each side of the channel is likely influenced by a species' ability to fly, we used wing morphology as a proxy for this trait. We classified species as macropterous (long winged, i.e., able to fly), brachypterous (short winged/wingless, i.e., unable to fly) or polymorphic (some individuals may be able to fly) using Luff (2007) and Knapp et al. (2020). We then produced three datasets, one including all species (hereafter, the *all‐species* dataset), one including macropterous species (the *flight‐capable* dataset), and one including only brachypterous and polymorphic species (the *limited‐flight* dataset).

We used Sørensen distances to represent community dissimilarity, i.e., β diversity, between assemblages in each dataset. Sørensen distances were used because pitfall trapping characterises activity densities, not abundance, thus reducing the reliability of abundance‐weighted measures (Engel et al. 2017). We calculated Sørensen β diversity from species occurrence in each dataset using the betapart package (Baselga et al. 2021). We then extracted the β diversity value comparing assemblages from the left and right bank for each site and visit. After preliminary analysis, we removed both B3 V4 samples (Figure 1a), because the right bank sample contained only one *Trechus obtusus* , a taxon not otherwise recorded, inflating cross‐channel β diversity and biasing the results. As such, we analysed 92 samples.

To determine whether observed cross‐channel β diversity deviated from that expected under random community assembly, we implemented a null model randomisation approach. We generated 999 randomised community matrices via the permatswap function in the vegan package (Oksanen et al. 2019), preserving row and column sums to maintain species occurrence frequencies and sample richness. For each randomised matrix, we then repeated the extraction of β diversity values comparing left and right bank samples for each site and visit. We used the extracted values to calculate the standardised effect size (z‐score) of cross‐channel β diversity as the difference between observed β diversity and the null mean, divided by the null standard deviation. Negative and positive z‐scores indicate that cross‐channel communities are less and more dissimilar than expected by chance, respectively.

To test H1–2, we used z‐scores as response variables in linear mixed‐effect models (LMM) built using the lmerTest package (Kuznetsova et al. 2017). We used longitudinal position (sites ranked from downstream to upstream: 1–6) and visit number (1–4) as fixed effects. We accounted for the non‐independence of samples collected at the same site on multiple visits by nesting site within catchment as a random intercept. To identify how communities differed between longitudinal positions or visits, we partitioned cross‐channel β diversity into its turnover (i.e., replacement) and nestedness‐resultant (i.e., richness difference) components using the betapart package (Baselga et al. 2021). We then replicated our null model approach to determine the contribution of each component to differences between longitudinal positions or visit numbers. For all LMMs, we distinguished the variance explained by the fixed and random factors using marginal (R^2^M) and conditional (R^2^C) R^2^, calculated using the MuMIn package (Bartoń 2020). We tested all model assumptions using the DHARMa package (Hartig 2020). We conducted all analyses in R v.4.0.3 (R Core Team 2020). We used *Pantheon* (Webb et al. 2018), a national database detailing the habitat preferences of each ground beetle species, to support out interpretations.

## 3 Results

### 3.1 In‐Channel Conditions

On the first visit (V1), both rivers were flowing at all sites. Gauged discharge decreased from 6.4 ± 0.2 m^3^/s (mean ± SD) and 0.7 ± < 0.1 m^3^/s during V1 to 5.1 ± 0.2 m^3^/s and 0.5 ± < 0.1 m^3^/s during V4 in the Bourne Rivulet and Candover Brook, respectively (Figure 2). Consistent with decreasing discharge in these downstream reaches, we observed a gradient of decreasing water width and depth at all sites with each successive visit, and the uppermost site (6) on each river dried between V3 and V4. Between V1 and V4, dense herbaceous vegetation encroached from the channel margins towards the channel centre at all sites (Figures S1–S4), slowing flow velocities and limiting solar radiation at the water's surface and channel bed.

### 3.2 Community Summary

**FIGURE 3:**
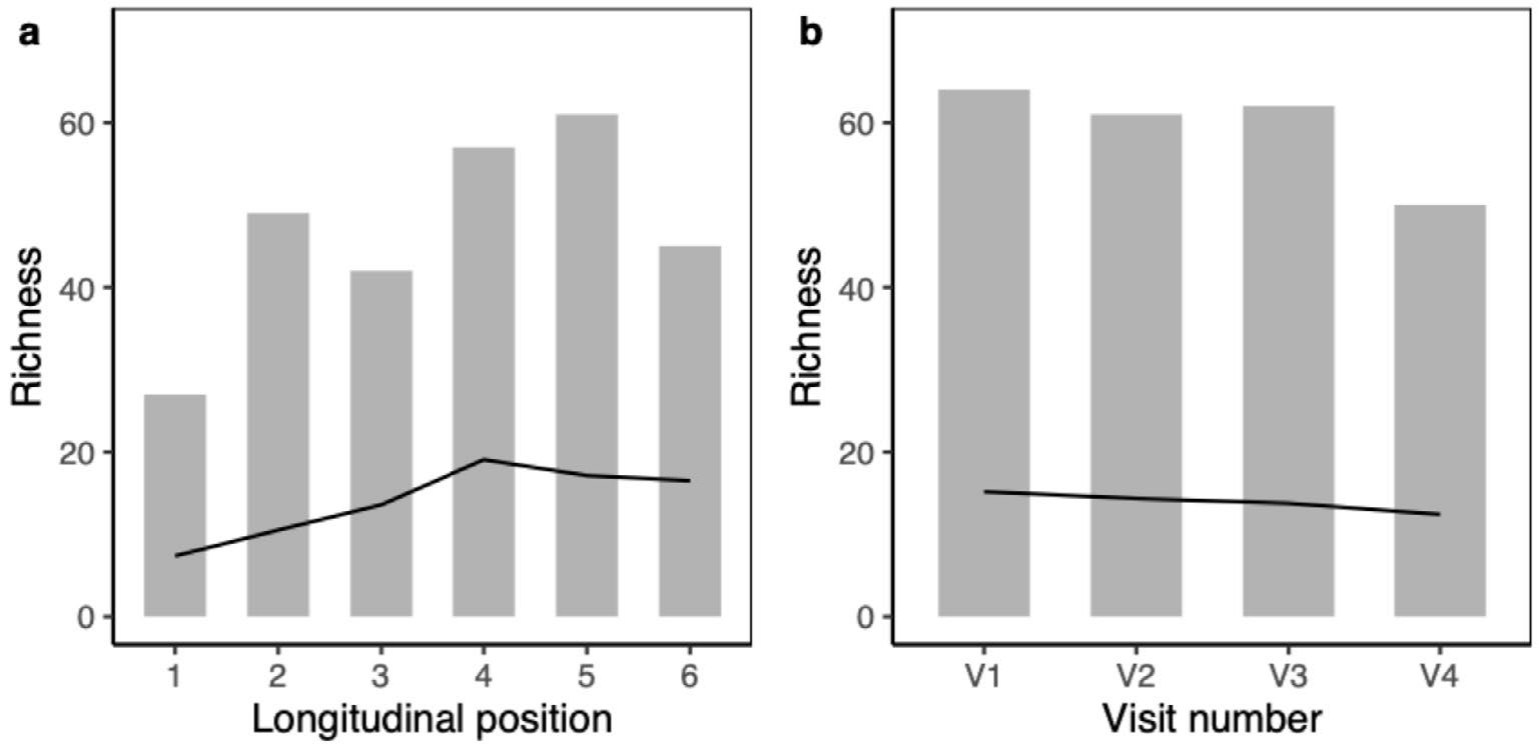
Total (bar) and mean per sample (line) richness of ground beetles captured at each longitudinal position (a) and during each sampling visit (b).

We collected 4236 ground beetles from 82 species, with samples containing 10.5 ± 5.1 (mean ± SD) species and 46.0 ± 41.9 individuals (Figure 3, Table S2). *Nebria brevicollis* (494 individuals), *Agonum emarginatum* (441) and *Pterostichus nigrita/rhaeticus* (378) were captured most frequently. We recorded 22 species only on one bank, all of which were infrequently captured (2.8 ± 3.2 individuals, range: 1–12). Most captured species were macropterous (64.6%), followed by polymorphic (24.4%) and brachypterous (11.0%). The flight‐capable dataset contained 2888 individuals representing 53 species; samples contained 6.7 ± 3.8 species and 31.2 ± 33.9 individuals; and the three most abundant species were *N. brevicollis* , *A. emarginatum* and *P. nigrita/rhaeticus*, comprising 45.5% of captures. The limited‐flight dataset comprised 29 species and 1348 individuals; samples contained 3.9 ± 2.0 species and 14.7 ± 16.1 individuals; and the three most common species ( *P. madidus* , 312 individuals; *Pterostichus minor*, 258; *Bembidion tetracolum* , 187) accounted for 56.2% of captures.

**FIGURE 4:**
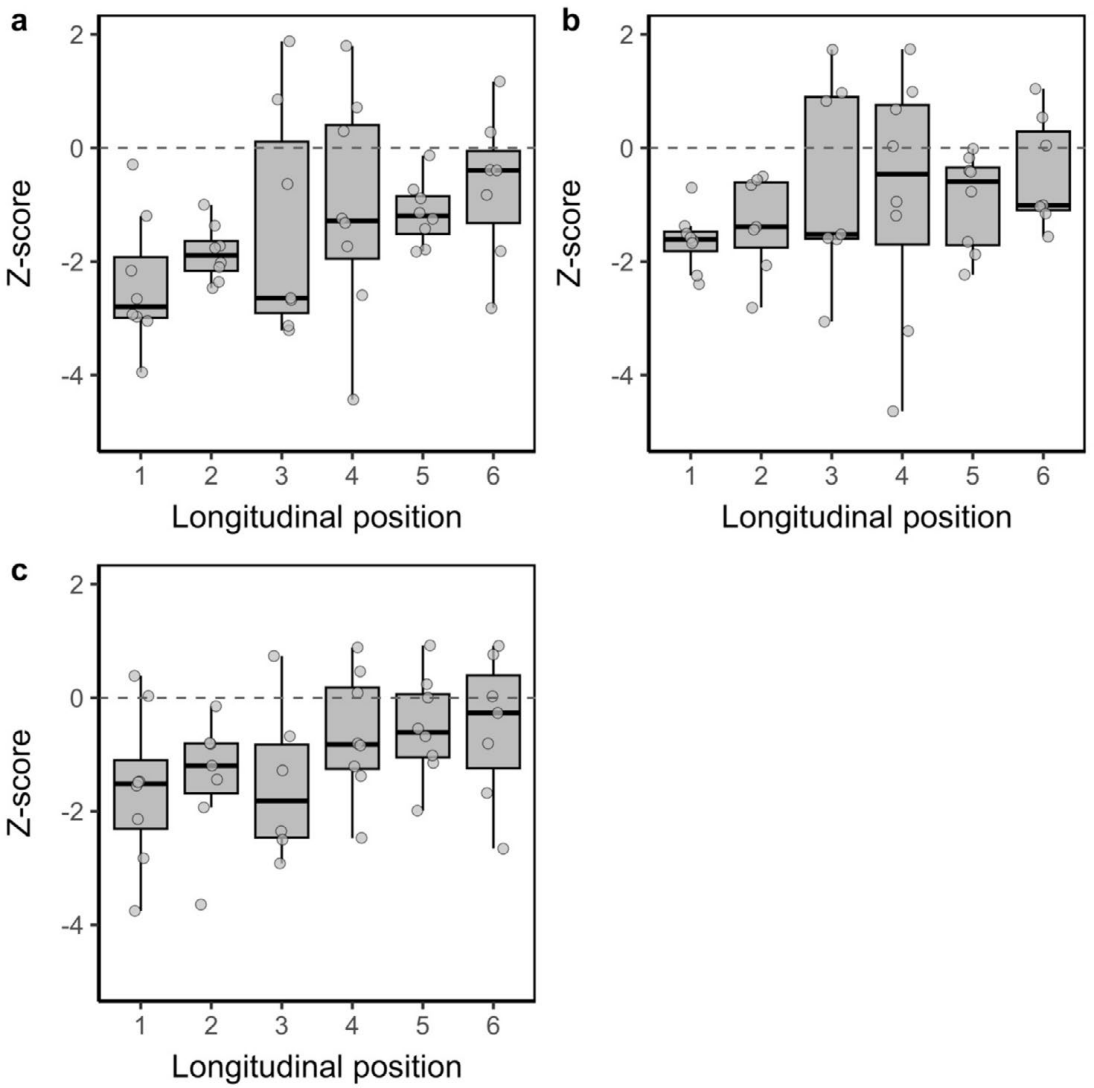
Standardised cross‐channel Sørensen β diversity (z‐scores) from sites at downstream (1) to upstream (6) longitudinal positions, for all‐species (a), flight‐capable (b) and limited‐flight (c) assemblages. The centre line represents the median, boxes represent the interquartile range, whiskers represent the minimum/maximum values which are within 1.5× the interquartile range of the first and third quartiles, and circles represent outliers.

**FIGURE 5:**
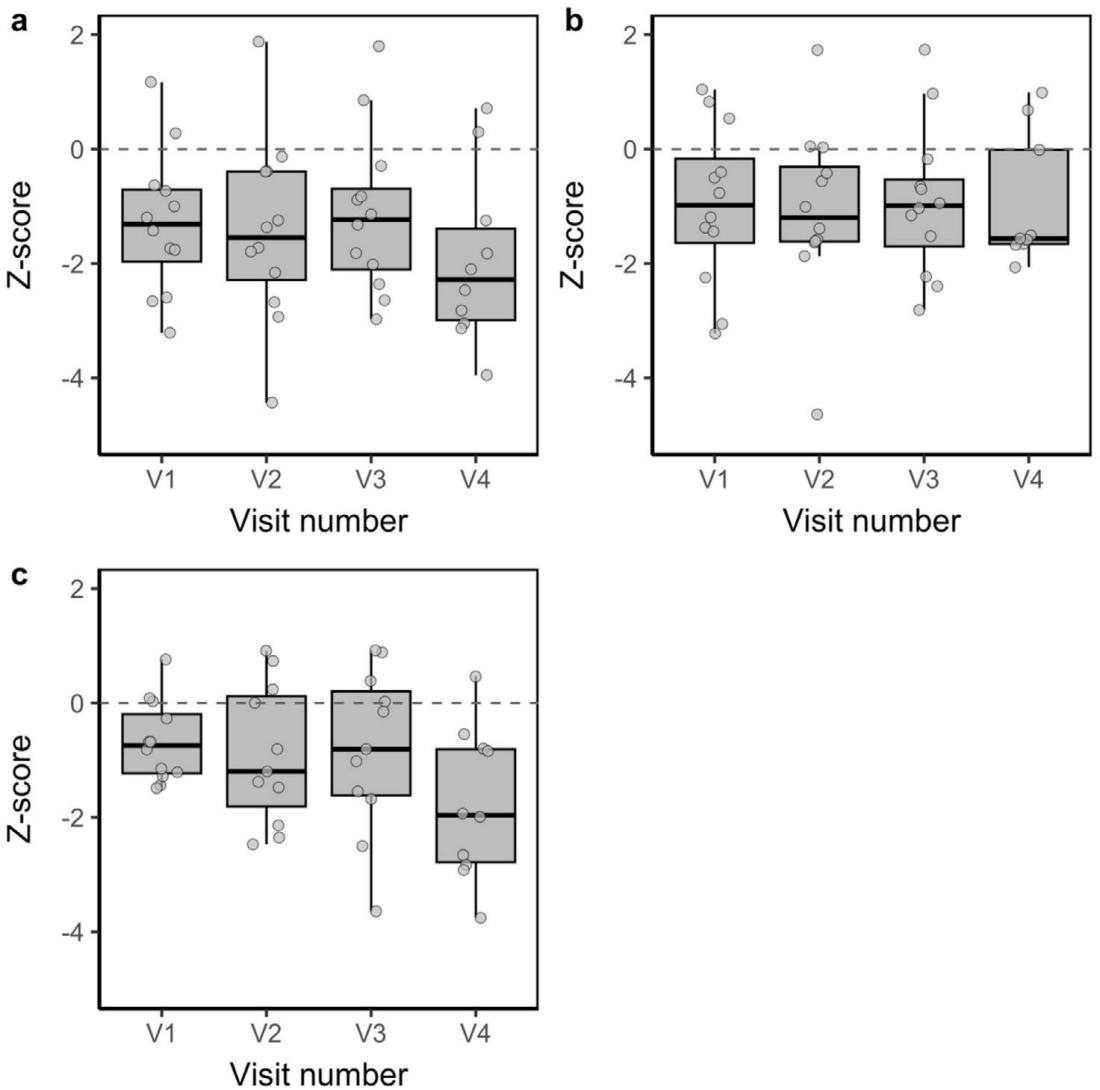
Standardised cross‐channel Sørensen β diversity (z‐scores) for assemblages sampled on visits (V) 1–4 in the all‐species (a), flight‐capable (b) and limited‐flight (c) datasets. The Figure 4 legend provides further details.

Cross‐channel communities had comparable Sørensen β diversity across all three datasets (Table 1), and all had lower β diversity than expected by chance (i.e., z‐scores were negative: intercept ± SE = −2.239 ± 0.725 to −1.158 ± 0.538, *p* = 0.006–0.037, R^2^M = 0.062–0.199, R^2^C = 0.199–0.360: Figures 4 and 5).

**TABLE 1:** Summary statistics (minimum, mean, standard deviation and maximum) of cross‐channel Sørensen β diversity for the all‐species, flight‐capable and limited‐flight datasets.

Dataset	Min	Mean	SD	Max
All‐species	0.167	0.549	0.184	1.000
Flight‐capable	0.000	0.542	0.212	1.000
Limited‐flight	0.000	0.583	0.256	1.000

### 3.3 H1: Cross‐Channel β Diversity Along a Drying Gradient

Contrary to H1, longitudinal position did not influence the cross‐channel β diversity of all‐species (estimate ± SE = 0.312 ± 0.156, *p* = 0.075, R^2^M = 0.140, R^2^C = 0.360: Figure 4a) or flight‐capable (estimate ± SE = 0.192 ± 0.157, *p* = 0.251, R^2^M = 0.062, R^2^C = 0.334: Figure 4b) assemblages, although cross‐channel β diversity tended to increase from downstream to upstream (Figure 4). Also contrary to H1, the cross‐channel β diversity of limited‐flight assemblages increased from downstream to upstream (estimate ± SE = 0.246 ± 0.097, *p* = 0.016, R^2^M = 0.199, R^2^C = 0.199: Figure 4c), due to turnover (estimate ± SE = 0.246 ± 0.097, *p* = 0.016, R^2^M = 0.199, R^2^C = 0.199), not nestedness‐resultant dissimilarity (estimate ± SE = −0.159 ± 0.105, *p* = 0.139, R^2^M = 0.142, R^2^C = 0.142).

### 3.4 H2: Cross‐Channel β Diversity Over Time

Cross‐channel β diversity was comparable among sampling visits for the all‐species (estimate ± SE = −0.102 ± 0.160, *p* = 0.528: Figure 5a) and flight capable (estimate ± SE = 0.079 ± 0.157, *p* = 0.617: Figure 5b) assemblages, contrary to H2. For the limited‐flight assemblage, cross‐channel β diversity was near‐significantly lower on V4 than on earlier visits (estimate ± SE = −0.298 ± 0.151, *p* = 0.055). *P. madidus*, the most common species in the limited‐flight dataset, increased in abundance and dominance from V1 (5 individuals, 1.4% of captures) to V2 (28, 11.2%), V3 (43, 12.8%) and V4 (236, 57.8%). During V1, we captured *P. madidus* on one bank at three sites on one river (C2, C4 and C6), whereas during V4, all sites except C1 and B1 supported *P. madidus* and, at the 10 sites at which it was captured, it occurred on both banks except at B2 and B3. We observed a similar pattern throughout the limited‐flight dataset, with 59 occurrences of a species on only one bank during V1, reducing to 35 such occurrences during V4.

Species with specific habitat preferences (e.g., *Paranchus albipes* , which has an affinity for running water) or resource requirements (e.g., *Badister unipustulatus* and *B. tetracolum* , which are snail and aquatic‐insect hunters, respectively) were notable exceptions to the pattern of increasing abundance over time. Between V1 and V4, captures of *P. albipes* , *B. unipustulatus* and *B. tetracolum* decreased from 75, 20 and 85 individuals to 4, 1 and 2 individuals, respectively. During V1, 60%, 50% and 50% of sites supporting *P. albipes* , *B. unipustulatus* and *B. tetracolum* did so on both banks, whereas no site supported these species on both banks during V4. Between V1 and V4, 24 species, 10 of which are associated with wetland habitats, were lost (Table S3). Over the same period, 10 species were gained, nine of which are associated with open habitats and trees and one (*Bembidion dentellum*) was wetland associated (Table S3).

## 4 Discussion

We demonstrate that the cross‐channel β diversity of ground beetle species that are capable of flight was spatiotemporally comparable along a drying gradient, suggesting that in‐channel conditions do not limit their movement across rivers, contrary to H1 and H2. In contrast, cross‐channel β diversity of species with limited flight capabilities increased along the longitudinal drying gradient, contrary to H1 and suggesting that the extent of surface water structures such assemblages. We observed decreases in cross‐channel β diversity over time as water levels declined, suggesting that decreasing water widths and depths, and the growth of extensive, channel‐spanning vegetation, also increased lateral connectivity between assemblages of non‐flying taxa on parallel banks. These patterns indicate that dispersal mechanisms, whether aerial, aquatic or ground‐based, interact with in‐channel conditions to shape community cross‐channel composition.

### 4.1 H1: Cross‐Channel β Diversity Along a Drying Gradient

Although many invertebrate taxa avoid crossing linear features (e.g., roads: Mader 1984; Andersson et al. 2017), cross‐channel β diversity was comparable along the drying gradient for all‐species and flight‐capable assemblages, contrary to H1. This may reflect that species capable of flight can cross rivers more easily while avoiding unfavourable in‐channel conditions (e.g., flowing water, elevated predation risk, higher temperatures: Lindroth 1992; Brose 2003; Steward 2012; Langhans and Tockner 2015). Additionally, over the spring/summer study period, vegetation encroached into the channels and flow velocities declined along the gradient. Such changes may have facilitated movement between parallel riparian zones regardless of water levels by increasing the proportion of individuals crossing the channel by swimming (Kolesnikov et al. 2012), climbing on vegetation (Hannam et al. 2008; Riddick 2008) or rafting on floating material (Corti and Datry 2012; Fleming et al. 2021). Thus, flight‐capable invertebrates in particular may perceive wet and dry river channels as similarly weak barriers to movement, fostering comparable cross‐channel β diversity along the drying gradient.

For limited‐flight assemblages, cross‐channel β diversity increased from downstream to upstream, contrary to H1. This trend likely reflects the environmental heterogeneity caused by transitions between wet and dry phases at our upstream sites (Datry et al. 2016; Ruhí et al. 2017). Thus, our most temporally heterogenous sites supported more variable cross‐channel communities, highlighting community variability both between (Moody and Sabo 2017) and within sites. Beta diversity along flow permanence gradients can be increased by unique, site‐specific species (Moody and Sabo 2017; Sánchez‐Montoya et al. 2020). Accordingly, we observed a general increase in richness and turnover from downstream to upstream, suggesting that declining water levels and ultimately river drying drive greater cross‐channel β diversity (consistent with Finn et al. 2011; Kabir et al. 2024).

The downstream‐to‐upstream increase in β diversity was observed in all datasets but only significant for limited‐flight taxa, suggesting that water is a driver of, and not a barrier to, movement. Aquatic food sources (e.g., Paetzold et al. 2005; Greenwood and McIntosh 2010; Gething 2025b) and species‐specific inundation tolerances (Eyre, Luff, and Phillips 2001; Bates et al. 2007; O'Callaghan et al. 2013) likely influence the lateral and longitudinal distribution of riparian arthropods (Ružanović et al. 2025). For example, some brachypterous *Bembidion* species are common in riparian habitats (D. A. Lott 2003; Luff 2007) and are strong swimmers (Joy 1910; Andersen 1968) that feed on emerging aquatic insects (Hering and Plachter 1997). The presence rather than absence of water may influence the cross‐channel distribution of such species because, although the absence of water may facilitate ground‐based cross‐channel movements, the resource incentive to enter, or remain in, the channel is lost soon after drying (Lassau et al. 2005; Gething et al. 2022).

In temporary headwater reaches, falling water levels, drying sediments and encroaching vegetation often mean that channels increasingly resemble surrounding riparian habitats as dry‐phase durations extend. Such reaches typically support generalist riparian beetle species (Eyre, Lott, and Luff 2001; Eyre, Luff, and Phillips 2001), which have morphological and behavioural traits that enable movement through dense vegetation (e.g., *P. madidus*: Evans and Forsythe 1984; Evans 1994; Lott 2001), and which can exploit in‐channel resources during and immediately after drying (i.e., ‘clean‐up crews’ feeding on dying and dead aquatic organisms: Williams 2006; Steward et al. 2022; Gething 2025b). Once such resources are depleted, communities in and around the channel are likely to comprise a temporally variable subset of patchily distributed species from surrounding habitats (Moody and Sabo 2017; Gething 2025a) that are searching for resources or breeding partners (Lövei and Sunderland 1996; Matalin 2003; Lagisz et al. 2010), potentially accounting for the observed non‐significant increase in β diversity with distance upstream. Thus, the more persistent presence of water at downstream sites may have reduced cross‐channel β diversity by providing a relatively stable resource around which communities assembled (Lassau et al. 2005; Gething et al. 2022).

### 4.2 H2: Cross‐Channel β Diversity Over Time

Cross‐channel β diversity remained comparable for the flight‐capable and thus the macropterous‐species‐dominated all‐species assemblages as water levels declined, contrary to H2. This comparability likely reflects that species capable of flight are particularly able to move between banks regardless of in‐channel conditions and allowed them to cross the channel throughout the study period. In contrast, concurrent near‐significant declines in the cross‐channel β diversity of the limited‐flight assemblage and water levels offer some support for H2 and suggest that drying increased lateral connectivity between parallel riparian zones. Decreasing water levels and the ‘bridge’ formed by encroaching vegetation may have enabled cross‐channel movement of non‐flying species (Steward 2012; Langhans and Tockner 2015).

The increased occurrence and abundance of non‐flying species between V1 (April) and V4 (July) likely reflects a seasonal increase as such species spread from overwintering, hatching or larval habitats. For example, seasonal increases in *P. madidus* densities can trigger movement in search of resources (Desender et al. 1994; Lövei and Sunderland 1996), competitor‐free space (Lenski 1984; Loreau 1990) or egg‐laying sites (Rijnsdorp 1980; van Huizen 1984). The increased occurrence of such brachypterous species on both banks also suggests that concurrent declining flows allowed movement into parallel riparian zones. Thus, although in‐channel conditions likely determine the frequency of cross‐channel movements (Gething et al. 2026), resource availability, population dynamics and species‐specific life histories may also influence cross‐channel β diversity.

Species colonising from one bank typically replaced those on the newly colonised bank (i.e., decreases in cross‐channel β diversity were driven by turnover), consistent with reported changes in community composition during drying (McCluney and Sabo 2012; Corti and Datry 2014; Sánchez‐Montoya et al. 2016). Aquatic barriers to ground‐based movement decline during drying, which may allow generalist species such as *P. madidus* to colonise then outcompete more specialised species such as the snail‐hunter *B. unipustulatus*, potentially reducing the diversity of such specialists. Generalist species are often more common in upstream riparian communities (Eyre, Lott, and Luff 2001; Eyre, Luff, and Phillips 2001; Gething et al. 2026), where even flowing rivers are lesser barriers to movement. Thus, the temporal variability in environmental conditions at upstream sites may have favoured generalist species which can better tolerate changing conditions such as drying (Devictor et al. 2008; Datry et al. 2014). However, a decline in wetland‐associated species coincided with drying, and *B. unipustulatus* and *B. tetracolum* may also have declined in response to a loss of aquatic prey, consistent with the absence of aquatic‐resource‐reliant beetles alongside temporary reaches observed by Moody and Sabo (2017).

## 5 Conclusions

As river drying becomes more prevalent under shifting climatic conditions and increasing water resource demands (Zipper et al. 2021; Zhang et al. 2023; Mimeau et al. 2025), our study is the first to characterise how cross‐channel β diversity of riparian ground beetle communities responds to declining water levels and river drying. For species with limited flight capabilities, we found that declining flows increased cross‐channel connectivity, resulting in the replacement of specialised wetland species by generalists. Biodiversity could be supported by balancing the isolation needed to protect specialists from competitive generalists with the cross‐channel connectivity that communities require for long‐term resilience and by promoting access to key resources such as water. Management actions should thus seek to support flow regimes that mitigate climate‐driven shifts in the spatiotemporal extent of river drying.

## Author Contributions

**Kieran J. Gething:** conceptualization (lead), data curation (lead), formal analysis (lead), investigation (lead), methodology (lead), project administration (lead), visualization (lead), writing – original draft (lead), writing – review and editing (equal). **Romain Sarremejane:** conceptualization (supporting), formal analysis (supporting), methodology (supporting), writing – review and editing (equal). **Chloe Hayes:** conceptualization (supporting), writing – review and editing (equal). **Jaime Martin:** conceptualization (supporting), writing – review and editing (equal). **Robert I. Collier:** conceptualization (supporting), writing – review and editing (equal). **Jonathan R. Webb:** conceptualization (supporting), writing – review and editing (equal). **Judy England:** conceptualization (supporting), writing – review and editing (equal). **Tim Sykes:** conceptualization (supporting), investigation (supporting), writing – review and editing (equal). **Rachel Stubbington:** conceptualization (supporting), writing – original draft (supporting), writing – review and editing (equal).

## Funding

This study was supported by Nottingham Trent University, the Environment Agency, Hampshire and Isle of Wight Wildlife Trust and the Vitacress Conservation Trust.

## Conflicts of Interest

The authors declare no conflicts of interest.

## Supporting information


**Data S1:** ece373568‐sup‐0001‐Supinfo.docx.

## Data Availability

Data available from: https://doi.org/10.5061/dryad.xpnvx0kwp, code available from: https://doi.org/10.5281/zenodo.19710712.
