# Supplementary material for: Fly or Dry? River Flow and Dispersal Mode Drive Cross‐Channel β Diversity in Riparian Zones
Source: Ecol Evol. 2026 May 4;16(5):e73568. doi: 10.1002/ece3.73568 (PMC13139725; doi:10.1002/ece3.73568)
Supplement: Supplementary file 1 — Data S1: ece373568‐sup‐0001‐Supinfo.docx. [file ECE3-16-e73568-s001.docx]

**Fly or dry? River flow and dispersal mode drive cross-channel β diversity in riparian zones**

Kieran J. Gething, Romain Sarremejane, Chloe Hayes, Jaime Martin, Robert I. Collier, Jonathan R. Webb, Judy England, Tim Sykes and Rachel Stubbington

**Correspondence:** Kieran J. Gething (https://orcid.org/0000-0002-4997-0249)

**Email:** k.gething@lboro.ac.uk


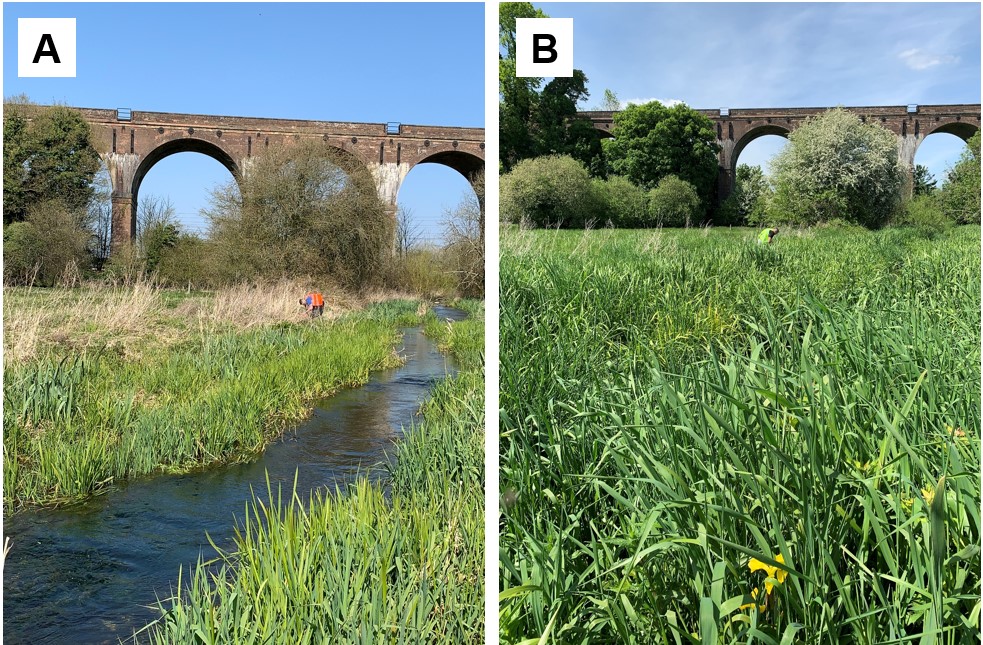


**Figure S1.** Site B1 on the Bourne Rivulet during the first (A) and last (B) sampling visit.


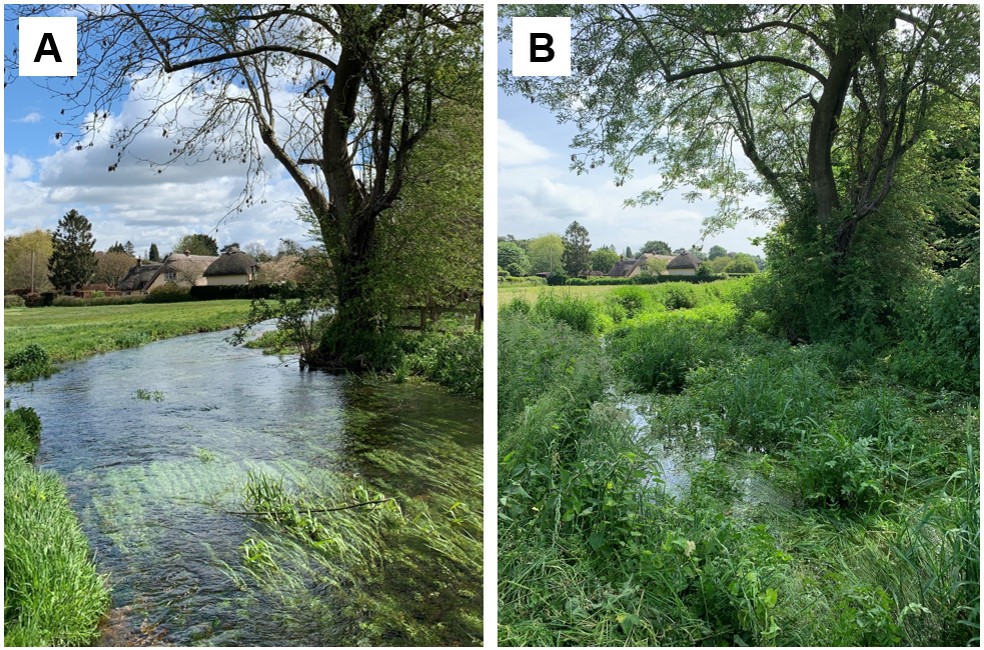


**Figure S2.** Site B4 on the Bourne Rivulet during the first (A) and last (B) sampling visit.


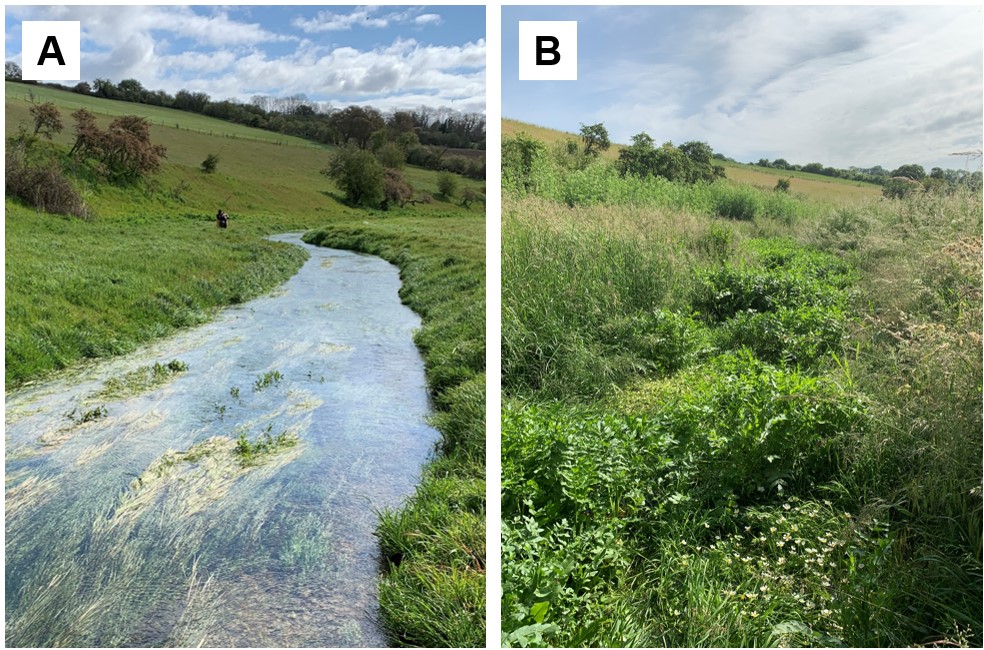


**Figure S3.** Site B6 on the Bourne Rivulet during the first (A) and last (B) sampling visit.


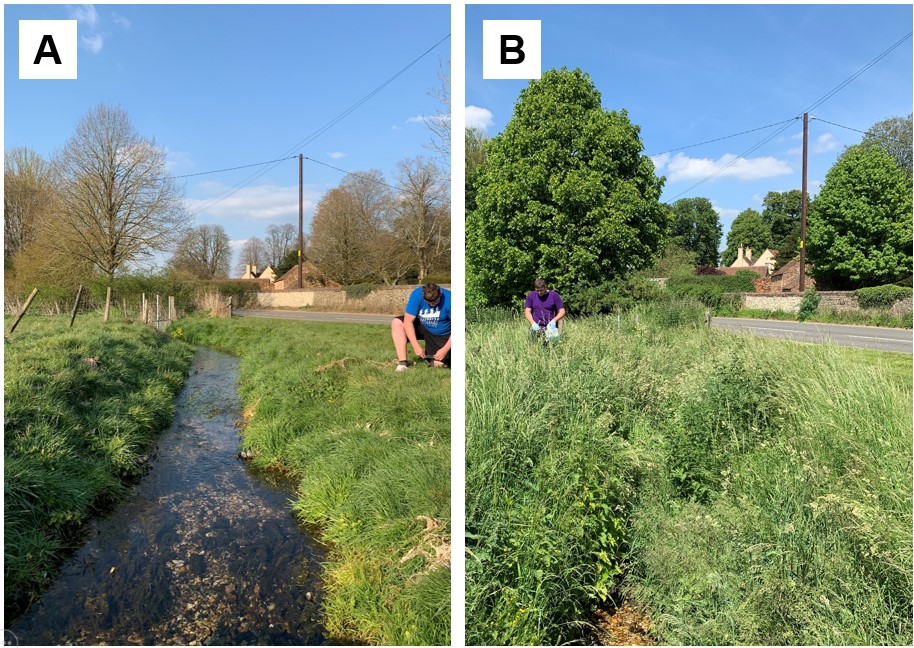


**Figure S4.** Site C6 on the Candover Brook during the first (A) and last (B) sampling visit.

**Table S1.** Summary of left and right-bank land use and bank slope for sampling sites in the Bourne Rivulet (B) and Candover Brook (C) catchments.

| **Site** | **Land use: left** | **Land use: right** | **Bank slope (°): left** | **Bank slope (°): right** |
| --- | --- | --- | --- | --- |
| **B1** | Wetland | Wetland | 20 | 20 |
| **B2** | Mown grassland | Woodland | 40 | 40 |
| **B3** | Mown grassland | Mown grassland | 40 | 40 |
| **B4** | Mown grassland | Mown grassland | 20 | 20 |
| **B5** | Mown grassland | Mown grassland | 30 | 30–90 |
| **B6** | Pasture | Pasture | 30 | 30 |
| **C1** | Pasture | Pasture | 20 | 20 |
| **C2** | Wetland | Wetland | 20 | 20 |
| **C3** | Pasture | Mown grassland | 20 | 20 |
| **C4** | Pasture | Pasture | 40 | 40 |
| **C5** | Wet woodland | Mown grassland | 20 | 35 |
| **C6** | Mown grassland | Pasture | 40 | 40 |

**Table S2.** The number of species captured per sample in the Bourne Rivulet and Candover Brook catchments, in temporary and perennial reaches and during visits (V) 1–4.

|  | **Minimum** | **Mean ± SD** | **Maximum** |
| --- | --- | --- | --- |
| **Bourne Rivulet** | 0 | 9.8 ± 6.0 | 22 |
| **Candover Brook** | 3 | 10.9 ± 4.4 | 20 |
| **V1** | 4 | 11.7 ± 5.9 | 20 |
| **V2** | 1 | 10.8 ± 5.4 | 22 |
| **V3** | 2 | 10.3 ± 5.5 | 20 |
| **V4** | 0 | 8.5 ± 5.1 | 21 |

**Table S3.** The abundance of species gained and lost between visits (V) 1–4, their habitat association and flight ability.

| **Species** | **Habitat** | **Flight ability** | **V1** | **V2** | **V3** | **V4** |
| --- | --- | --- | --- | --- | --- | --- |
| *Acupalpus parvulus* | Wetlands | Macropterous | 1 | 0 | 1 | 0 |
| *Agonum fuliginosum/gracile* | Wetlands | Polymorphic | 12 | 0 | 0 | 0 |
| *Agonum piceum* | Wetlands | Polymorphic | 3 | 0 | 0 | 0 |
| *Amara aenea* | Open areas | Macropterous | 8 | 1 | 0 | 0 |
| *Amara familiaris* | Open areas | Macropterous | 2 | 1 | 0 | 0 |
| *Anisodactylus binotatus* | Wetlands | Macropterous | 1 | 1 | 2 | 0 |
| *Asaphidion stierlini* | Open areas | Macropterous | 1 | 0 | 0 | 0 |
| *Badister peltatus* | Wetlands | Macropterous | 1 | 1 | 0 | 0 |
| *Bembidion assimile* | Wetlands | Polymorphic | 11 | 1 | 2 | 0 |
| *Bembidion varium* | Wetlands | Macropterous | 1 | 0 | 0 | 0 |
| *Bembidion biguttatum* | Wetlands | Macropterous | 3 | 3 | 0 | 0 |
| *Bembidion guttula* | Wetlands | Polymorphic | 8 | 4 | 5 | 0 |
| *Bembidion obtusum* | Open areas | Polymorphic | 6 | 0 | 1 | 0 |
| *Curtonotus aulicus* | Open areas | Macropterous | 3 | 0 | 0 | 0 |
| *Harpalus rubripes* | Open areas | Macropterous | 1 | 1 | 5 | 0 |
| *Leistus fulvibarbis* | Tree-associated | Macropterous | 16 | 6 | 2 | 0 |
| *Leistus terminatus* | Open areas | Brachypterous | 2 | 0 | 0 | 0 |
| *Microlestes minutulus* | Open areas | Macropterous | 1 | 0 | 0 | 0 |
| *Notiophilus rufipes* | Tree-associated | Macropterous | 2 | 0 | 0 | 0 |
| *Oxypselaphus obscurus* | Tree-associated | Brachypterous | 8 | 3 | 2 | 0 |
| *Paradromius linearis* | Open areas | Polymorphic | 2 | 0 | 0 | 0 |
| *Pterostichus vernalis* | Open areas | Polymorphic | 1 | 0 | 0 | 0 |
| *Stenolophus mixtus* | Wetlands | Macropterous | 5 | 1 | 1 | 0 |
| *Syntomus foveatus* | Open areas | Brachypterous | 12 | 2 | 12 | 0 |
| *Abax parallelepipedus* | Woodland floor | Brachypterous | 0 | 0 | 3 | 1 |
| *Amara plebeja* | Open areas | Macropterous | 0 | 0 | 1 | 2 |
| *Bembidion quadrimaculatum* | Open areas | Macropterous | 0 | 0 | 1 | 1 |
| *Bembidion dentellum* | Wetlands | Macropterous | 0 | 2 | 0 | 1 |
| *Calathus rotundicollis* | Tree-associated | Polymorphic | 0 | 1 | 1 | 1 |
| *Carabus violaceus* | Open areas | Brachypterous | 0 | 0 | 8 | 4 |
| *Harpalus rufipes* | Open areas | Macropterous | 0 | 13 | 9 | 14 |
| *Harpalus tardus* | Open areas | Macropterous | 0 | 0 | 0 | 1 |
| *Pterostichus niger* | Open areas | Macropterous | 0 | 0 | 2 | 2 |
| *Pterostichus oblongopunctatus* | Tree-associated | Macropterous | 0 | 2 | 0 | 1 |
